# Mannooligosaccharide production from açaí seeds by enzymatic hydrolysis: optimization through response surface methodology

**DOI:** 10.1007/s11356-024-33540-2

**Published:** 2024-06-12

**Authors:** Sarha Lucia Murillo-Franco, Juan David Galvis-Nieto, Carlos E. Orrego

**Affiliations:** 1 Departamento de Ingeniería Química, Instituto de Biotecnología y Agroindustria, Universidad Nacional de Colombia, 170003 Manizales, Caldas, Colombia; 2 São Paulo State University (UNESP), Institute of Chemistry, Araraquara, São Paulo, 14800-900 Brazil; 3 Departamento de Física y Química, Instituto de Biotecnologia y Agroindustria, Universidad Nacional de Colombia, 170003 Manizales, Caldas, Colombia

**Keywords:** *Euterpe oleracea*, Biocatalysis, Mannanase, Valorization, MOS, Surface response design, Optimization, Agro-food residues

## Abstract

Recognized for its bioactive compounds, açaí has become a functional food, but it has a low pulp yield, and the seeds are the main waste. This study investigates the potential of açaí seeds (*Euterpe oleracea* Mart.) to produce mannooligosaccharides (MOS) through enzymatic hydrolysis. Using response surface methodology (RSM), the research optimizes MOS extraction while minimizing mannose production and reducing processing time, achieving MOS production of about 10 g/L, a value within the range of similar investigations. The RSM quadratic models establish correlations between MOS production (M2–M5) and enzymatic hydrolysis conditions, with R2 values ranging from 0.6136 to 0.9031. These models are used to emphasize MOS performance (M2–M5) while reducing mannose production, which also promotes profitability by reducing time. Experimental validation agrees with model predictions, highlighting optimal conditions near 40 °C, intermediate enzyme loading, and basic pH that effectively promotes MOS generation on mannose within an accelerated processing time frame. With predictions of experimental results within a margin of error of < 9%, the validity of the models was acceptable. This research contributes to the advancement of the understanding of the enzymatic hydrolysis of açaí seeds, which is a step toward the sustainable use of resources with a focus on process engineering aspects.

## Introduction

The açaí palm (*Euterpe oleracea* Mart.) is native to extensive populations in the tropical and floodable regions of South America (Rojano et al. 2011). It has gained recognition as a functional food due to its abundance of bioactive compounds (Matta et al. 2020). This distinctive fruit has sparked considerable global interest in its commercialization. Remarkably, its primary exporter, Brazil, has witnessed a nearly 90% increase in açaí production between 2010 and 2020. In 2021, the annual production exceeded 1 million tons (SIDRA 2021). Similarly, various entities in Colombia have been actively promoting endeavors to bolster açaí production (MinAmbiente 2023), resulting in an annual yield of over 20,000 tons in 2020 (Agronet 2021).

The production of açaí pulp yields efficiencies of no more than 15% of the fruit, with the seed constituting the highest mass fraction and contributing the greatest environmental burden (Pessoa et al. 2010). These seeds can serve as raw material for renewable biomass to produce numerous biobased chemicals. The array of valorization alternatives includes utilization for biofuel production such as ethanol (Cordeiro et al. 2019) and biogas (Maciel-Silva et al. 2019), extraction of antioxidant (Martins et al. 2020; Melo et al. 2021; Viganó et al. 2022), and the generation of activated carbon (de Sousa Ribeiro et al. 2018; Sato et al. 2020). Furthermore, a study by Monteiro et al. (2019) found that mannan is the predominant component in açai seeds. It accounts for approximately 80% of the total sugar content and 50% of the dry weight. On the other hand, our research group found that açaí seeds contain 37.88% hemicellulose per gram of dry weight, with mannan being included in this value (Murillo-Franco et al. 2023).

The potential of açaí as a rich source of mannose has been explored, utilized in the production of β-endomannanase through solid-state fermentation (Lima et al. 2021). Moreover, Otieno and Ahring (2012) suggest that a material is deemed suitable for oligosaccharide production when it contains at least 20% of the desired monosaccharide. This characterization positions açaí seeds as ideal candidates for mannooligosaccharide (MOS) extraction. MOS presents an emerging prebiotic alternative with promising bioactive attributes (Jana et al. 2021). MOS has been shown to be effective as an animal supplement, promoting growth and reducing antibiotic use in pigs (Yu et al. 2021) and fish (Mo et al. 2015). While commercial MOS is currently extracted from various yeast sources, alternative options such as agro-food residues like copra meal and palm kernel cake have been investigated and have exhibited prebiotic, antioxidant, and anticarcinogenic potential (Jana and Kango 2020). These findings highlight their promising prospects as additives for the food and animal feed industries, demonstrating the potential of agro-food residues as a valuable source for MOS extraction.

Enzymatic hydrolysis is used to produce MOS from lignocellulosic materials, including palm kernel cake (Li et al. 2018), copra meal (Intaratrakul et al. 2022), and spent coffee grounds (Ghosh et al. 2015). A recent study also explored MOS production from açaí seeds (Murillo-Franco et al. 2023), but only under standard conditions.

To achieve efficient and economically viable MOS production, it is imperative to optimize multiple process variables. An effective avenue in this direction is the application of statistical experimental design, such as response surface methodology (RSM). This approach enables not only the evaluation of individual process variables impacts but also the comprehension of their interactions and combined effects (Myers et al. 2016).

In this context, the present study aims to position açaí seeds as a promising source for MOS extraction, utilizing an enzymatic hydrolysis approach. The objective is to determine the process variables that exert significant influence over MOS production. Based on the model results, the study seeks to correlate actual and adjusted yields for both MOS individually (M2–M5) and mannose, validating the model using the optimization of variables for maximum production of MOS and minimizing the mannose yield.

## Materials and methods

### Materials and reagents

The seeds utilized in this study were provided by Refrescos del Litoral Ltda., a company located in Quibdo, Chocó, Colombia. All the chemicals, buffers, and reagents employed in this research were of analytical grade. The procurement source for these chemicals was primarily Sigma Chemical Co. (USA), unless explicitly mentioned. The standards for mannooligosaccharides (including manobiose (M2), mannotriose (M3), mannotetraose (M4), and mannopentaose (M5)) were procured from Megazyme (Ireland). The enzyme utilized in the experiment was Rohalase® GMP, an enzymatic preparation sourced from AB Enzymes (Germany), with mannanase as its main activity.

### Pretreatment raw material

The seeds were washed thoroughly with water to remove any remaining fruit particles. Additionally, the fibrous part that covers the seed was manually removed. Subsequently, they were stored in a light-protected environment at a temperature lower than 0 °C, adhering to a time frame of no longer than 14 days, leading up to the analysis phase.

For the analysis, the seeds underwent a drying process at a temperature of 70 °C for a duration of 24 h. Post-drying, they were finely pulverized utilizing a mesh knife mill (Model TE-650/1 Tecnal, SP, Brazil) and then sifted through of set of US Standard Testing Sieves to achieve particle sizes no larger than 250 μm. This preparation ensured the creation of uniform suspensions, which is imperative for the subsequent hydrolysis procedure.

### Experimental design and procedure

The hydrolysis parameters were optimized through a Box-Behnken experimental design (BBD), executed with an approach involving five replications at the central point, enabling the estimation of the standard error of the experiments. A total of 29 experiments were conducted, encompassing an evaluation of three levels (low, neutral, and high) for each of the four independent variables: temperature (°C), pH, enzyme load (U/g substrate), and reaction time (h) (Table 1).

**Table 1:** Independent variables and their corresponding levels for mannooligosaccharide production

Independent variable	Unit	Low	Neutral	High
A, Time	hr	1	12.5	24
B, pH		5.5	7	8.5
C, Enzyme load	(U/g substrate)	17	33.5	50
D, Temperature	(°C)	30	50	70

Six response variables were recorded, representing the concentrations in milligrams per liter of distinct MOS (M2, M3, M4, M5) and mannose (M1). To ensure statistical validity, the sequence of experimental runs was randomized, thereby mitigating the potential impact of unforeseen variations in the observed responses. Subsequently, the empirical data generated from the Box-Behnken design were subjected to an analysis, employing the RSM (response surface methodology) algorithm implemented in Design Expert *V.13.0.5.0.* (Stat-Ease, Minneapolis, USA). This data was modeled and fitted using Eq. (1), representing a second-order polynomial equation (Eq. 1), in order to capture and understand the relationships between the various independent parameters and the resulting responses.

1 $${Y}_{i}={\beta }_{0}+{\sum }_{i=1}^{4}{\beta }_{i}{x}_{i}+\sum_{j=1}^{4}{\sum }_{i=1}^{4}{\beta }_{ij}{x}_{i}{x}_{j}+{\sum }_{i=1}^{4}{\beta }_{ii}{x}_{i}^{2}+\varepsilon$$

where ${\beta }_{i}$ symbolizes the linear effect attributable to ${x}_{i}$, ${\beta }_{ij}$ signifies the linear interaction between ${x}_{i}$, ${x}_{j}$, and ${\beta }_{ii}$ captures the quadratic effect stemming from ${x}_{i}$, and ${Y}_{i}$ denotes the respective response variable under consideration. Finally, three additional experiments corresponding to the optimized conditions were conducted to verify the validity of the statistical experimental strategies.

### Enzymatic hydrolysis for MOS production

The hydrolysates were prepared by mixing 3 g of powdered açaí seed with 30 mL of 0.05 M buffer solution. The pH of the buffer solutions varied depending on the type: pH 5.5 citrate buffer, pH 7.5 sodium phosphate buffer, and pH 8.5 boric acid-borate buffer were utilized. The preparation process ensured the maintenance of pH, enzymatic load, time, and temperature, in accordance with the predetermined values outlined by the Box-Behnken design. The hydrolysis reaction was stopped by boiling the samples for 5 min. Next, a 2-mL aliquot was taken from the reaction mixture and centrifuged at 13,500 rpm for 5 min at 9 °C. The resulting supernatant was collected and subjected to filtration using 0.2-µm nylon filters. This filtered solution was then transferred into amber glass vials. The vials were stored in a dark environment at a temperature of 4 °C for further analysis.

### Determination of MOS and mannose

For the assessment of mannooligosaccharide content within the hydrolysates, a high-performance liquid chromatography (HPLC) analysis was conducted using a Hitachi LaChrom Elite® HPLC System (Hitachi High Technologies America, Inc., USA), which was outfitted with a refractive index detector (RID L-2490). Separation was achieved using a Eurokat Ca column (10 µm, 300 × 4 mm) with water serving as the eluent at a flow rate of 0.15 mL/min. Operational parameters were set to 65 °C for the column temperature and 35 °C for the detector temperature. Quantitative analysis was based on peak areas in the chromatogram, correlating with calibration curves. These curves were established using mannose from Sigma Aldrich USA, as well as standards such as mannobiose (M2), mannotriose (M3), mannotetraose (M4), and mannopentaose (M5) from Megazyme, Ireland.

### Statistical analysis

The statistical analysis involved the utilization of analysis of variance (ANOVA) along with the generation of surface plots, both facilitated through the Design Expert software *V.13.0.5.0* (Stat-Ease, Minneapolis, USA). The significance of models and their terms was assessed via *p*-values at a 95% confidence level. The efficacy of the polynomial model was gauged using coefficients of determination, denoted as *R*^2^ and *R*^2^_(adj)_. To optimize parameters, an optimization package embedded within the same software was employed.

## Results and discussion

### Design and modeling

The degree of polymerization (DP) achieved in the assortment of generated MOS lends insight into the underlying chemical and biological attributes of these compounds. A pertinent example comes from Srivastava et al. (2017), who observed that employing M2 and M3 as carbon sources fosters more robust probiotic growth, as compared to the utilization of FOS or M5. Moreover, Kumar Suryawanshi and Kango (2021) also noted that MOS with shorter DP (< 4) exhibited enhanced inhibition of carcinogenic cells compared to the high-DP MOS.

Therefore, determine the optimal variables to ensure the production of M2–M5 oligosaccharide types of mandate control over operational parameters such as enzyme load, pH, temperature, and time. In this context, this study presents six RSM models aimed at elucidating the most important characteristics that exert the most significant influence on the generation of oligosaccharides with varying DP, ranging from M2 to M5 both individually and collectively, in addition to the M1 production. The manipulation of these variables and each set at designated levels was executed through a curated series of 29 randomized experimental iterations, as detailed in Table 2.

**Table 2:** Response surface design and response values of MOS and mannose production

Run	Time	pH	E/S	T°	M1	M2	M3	M4	M5	MOS (M2–M5)
	h		U/g	°C	mg/L	mg/L	mg/L	mg/L	mg/L	mg/L
1	1	8.5	33.5	50	270.00	1779.71	2660.99	1760.54	2830.82	9032.06
2	24	7	33.5	70	334.06	1646.27	3014.71	1630.77	2992.62	9284.37
3	12.5	7	33.5	50	768.51	3235.38	3764.58	1274.76	1793.69	10,068.40
4	12.5	5.5	50	50	1374.43	2372.89	4955.12	824.23	829.74	8981.98
5	24	7	33.5	30	1192.02	1733.40	4377.35	1077.30	1381.81	8569.86
6	1	7	50	50	928.25	2558.85	2610.56	548.13	745.57	6463.11
7	12.5	8.5	50	50	375.36	2082.64	4887.62	1562.51	1659.24	10,192.01
8	24	7	17	50	1378.11	4424.50	1571.77	1283.28	2079.72	9359.27
9	1	7	17	50	889.08	3243.78	2103.17	1352.23	1635.06	8334.24
10	12.5	7	33.5	50	570.39	2935.38	3214.58	1274.76	1776.69	9201.41
11	1	7	33.5	30	532.67	1706.07	3527.28	1358.92	1676.63	8268.90
12	12.5	7	50	70	771.20	1248.45	3563.93	1121.23	408.75	6342.36
13	12.5	7	33.5	50	687.81	2135.38	3514.58	1324.76	2363.69	9338.41
14	12.5	7	50	30	842.45	2605.44	4554.77	1128.56	892.26	9181.03
15	12.5	7	17	30	1149.33	2373.88	3891.71	934.53	894.92	8095.03
16	12.5	5.5	17	50	1142.73	3007.54	2952.97	1265.74	1363.75	8590.87
17	12.5	7	33.5	50	569.45	2485.38	4214.58	1174.76	2188.69	10,063.40
18	24	5.5	33.5	50	1250.47	3500.96	3599.52	1274.57	2160.86	10,535.90
19	12.5	5.5	33.5	30	986.39	1097.73	4593.08	1105.88	2139.50	8936.19
20	1	5.5	33.5	50	597.37	1721.61	3856.72	918.54	1404.88	7901.75
21	12.5	5.5	33.5	70	142.92	579.74	4598.94	1375.85	1083.09	7637.62
22	12.5	8.5	33.5	30	308.32	630.28	6416.37	1385.92	1856.75	10,289.30
23	24	7	50	50	1020.19	3678.14	3622.35	1083.03	1507.60	9891.12
24	12.5	8.5	17	50	1190.81	2449.28	2931.80	1175.35	2618.85	9175.28
25	1	7	33.5	70	671.79	923.61	3230.39	1383.20	1657.10	7194.35
26	24	8.5	33.5	50	139.26	1452.36	4512.58	1500.45	3519.53	10,984.90
27	12.5	7	17	70	1028.08	2231.29	3023.02	1469.61	2479.71	9203.63
28	12.5	8.5	33.5	70	904.65	398.68	4143.98	2143.69	2774.44	9460.79
29	12.5	7	33.5	50	542.24	2335.38	4114.58	1424.76	2043.69	9918.41

The related analysis of variance (ANOVA) concerning the models for each respective response variable is presented in Table 3. All the models were appropriately correlated using quadratic models. It is notable that correlations exceeding 60% exist between the correlation coefficient of the model and its corresponding adjusted value, indicative of an adequate alignment between these models and the empirical experimental data. Additionally, all the generated models yielded *p*-values below 0.05, highlighting their statistical significance. Other authors have reported that the utilization of quadratic models to correlate MOS (DP < 6) production through enzymatic hydrolysis is suitable, achieving *R*^2^ values ranging between 0.8984 and 0.9937 (Intaratrakul et al. 2022; Chen et al. 2013; Jian et al. 2013).

**Table 3:** Analysis of variance (ANOVA) for the response surface methodology (RSM) models for the six response variables

Response variable (mg/L)	*R*^2^	*R*^2^ adjusted	*p*-value
M1	0.8804	0.7608	0.0003
M2	0.9031	0.8806	< 0.0001
M3	0.8278	0.6136	< 0.0001
M4	0.8916	0.7833	0.0002
M5	0.9337	0.8674	< 0.0001
MOS (M2–M5)	0.8936	0.7873	0.0001

The regression polynomials obtained for each of the oligomers individually (M2, M3, M4, M5), mannose, and MOS (M2–M5) before eliminating non-significant terms are presented in equations Eq. (2)–Eq. (7) where *A*, *B*, *C*, and *D* correspond to time, pH, enzyme loading, and temperature, respectively. The response of each model is expressed in mg/L.

2 $$M1=2495.509+156.381\bullet A+2.670\bullet B-24.265\bullet C-72.783\bullet D-11.360\bullet A\bullet B-0.523\bullet A\bullet C-1.084\bullet A\bullet D-10.577\bullet B\bullet C+11.998\bullet B\bullet D+0.038\bullet C\bullet D+0.207\bullet {A}^{2}-16.74\bullet {B}^{2}+1.426\bullet {C}^{2}-0.038\bullet {D}^{2}$$

3 $$M2=-18362.784+178.531\bullet A+4574.913\bullet B-150.837\bullet C+298.205\bullet D-30.532\bullet A\bullet B-0.081\bullet A\bullet C+0.756\bullet A\bullet D+2.707\bullet B\bullet C+2.387\bullet B\bullet D-0.92\bullet C\bullet D+1.309\bullet {A}^{2}-328.360\bullet {B}^{2}+2.430\bullet {C}^{2}-3.065\bullet {D}^{2}$$

4 $$M3=13610.077-60.249\bullet A-3497.765\bullet B+148.312\bullet C-3.118\bullet D+30.562\bullet A\bullet B+2.033\bullet A\bullet C-1.158\bullet A\bullet D-0.468\bullet B\bullet C-18.985\bullet B\bullet D-0.093\bullet C\bullet D-5.770\bullet {A}^{2}+295.434\bullet {B}^{2}-1.893\bullet {C}^{2}-1.295\bullet {D}^{2}$$

5 $$M4=4645.884+16.339\bullet A-912.975\bullet B-3.300\bullet C-35.337\bullet D-8.929\bullet A\bullet B+0.796\bullet A\bullet C+0.575\bullet A\bullet D+8.370\bullet B\bullet C+4.065\bullet B\bullet D-0.411\bullet C\bullet D-0.217\bullet {A}^{2}+49.605\bullet {B}^{2}-0.758\bullet {C}^{2}+0.223\bullet {D}^{2}$$

6 $$M5=3963.135-98.839\bullet A-1803.569\bullet B+238.260\bullet C-8.759\bullet D-0.975\bullet A\bullet B+0.418\bullet A\bullet C+1.772\bullet A\bullet D-4.299\bullet B\bullet C+16.451\bullet B\bullet D-1.567\bullet C\bullet D+1.192\bullet {A}^{2}+106.143\bullet {B}^{2}-2.395\bullet {C}^{2}-0.654\bullet {D}^{2}$$

7 $$MOS (M2-M5)=3860.285+35.793\bullet A-1640.158\bullet B+232.352\bullet C+251.000\bullet D-9.874\bullet A\bullet B+3.166\bullet A\bullet C+1.945\bullet A\bullet D+6.319\bullet B\bullet C+3.485\bullet B\bullet D-2.990\bullet C\bullet D-3.485\bullet {A}^{2}+122.852\bullet {B}^{2}-2.616\bullet {C}^{2}-2.201\bullet {D}^{2}$$

The adequacy of each model was verified through a residual analysis (data not shown). The normal probability plot of the residuals exhibited linearity for all response variables, confirming the assumption of normality assumed for the models. Furthermore, in the respective plots of residuals versus predicted responses, a random dispersion was observed, indicating the reliability of the models.

The significance of each factor and their interactive effects are presented in Table 4. Each of the variables presented significance in some of the response variables to be evaluated, which indicates the great correlation that exists between each of the variables analyzed and the hydrolysis process to generate MOS individually and jointly in addition to mannose.

**Table 4:** Significance of the parameter of the models for each response

Coefficient	M1	M2	M3	M4	M5	MOS (M2–M5)
*Time*	0.0359^*^	0.0017^**^	0.073	0.2922	0.0014^**^	< 0.0001^**^
*pH*	0.0021^**^	0.0096^**^	0.4874	< 0.0001^**^	< 0.0001^**^	0.0028^**^
*E/S*	0.0315^*^	0.0161^*^	< 0.0001^**^	0.0247^*^	< 0.0001^**^	0.3618
*Temperature*	0.08	0.018^*^	0.001^**^	0.0006^*^	0.0157^*^	0.0353^*^
*Time* × *pH*	0.0441^*^	0.0073^**^	0.0204^*^	0.044^*^	0.902	0.525
*Time* × *E*/*S*	0.2813	0.9285	0.0765	0.0478^*^	0.5637	0.0374^*^
*Time* × *Temperature*	0.0138^*^	0.3183	0.2078	0.0781	0.0089^**^	0.109
*pH* × *E*/*S*	0.0104^*^	0.696	0.955	0.01^*^	0.441	0.559
*pH* × *Temperature*	0.0012^**^	0.6764	0.0135^*^	0.1016	0.0025^**^	0.6598
*E/S* × *Temperature*	0.8898	0.0922	0.8818	0.0717	0.0018^**^	0.002^**^
*Time*^*2*^	0.6996	0.2104	0.0003^**^	0.608	0.1567	0.0413^*^
*pH*^*2*^	0.5968	< 0.0001^**^	0.0009^**^	0.0605	0.0398^*^	0.1993
*E/S*^*2*^	< 0.0001^**^	0.0002^**^	0.0058^**^	0.002^**^	< 0.0001^**^	0.0037^**^
*Temperature*^*2*^	0.8322	< 0.0001^**^	0.0056^**^	0.1242	0.0263^*^	0.0007^**^

^*^: significant term *p*-value < 0.05; ^**^: significant term *p*-value < 0.01; without ^*^ or ^**^ indicates not significant term

In the hydrolysis process for mannose production, key parameters such as pH, time, and enzymatic load were identified as significant factors. Notably, pH exhibited a higher level of significance (*p*-value < 0.01) compared to the other two parameters (*p*-value < 0.05). Furthermore, significant interactions were observed between time and pH, as well as between time and temperature in the model (*p*-value < 0.05). Additionally, the quadratic enzymatic load also proved to be significant (*p*-value < 0.01). For M2 production, it was found that although all linear parameters were significant, time and pH had a more pronounced influence (*p*-value < 0.01), which was also reflected in their joint interaction (*p*-value < 0.01). However, this trend did not hold for quadratic parameters, as time did not emerge as significant in comparison to the others. For M3 production, the most significant parameters were temperature and enzymatic load (*p*-value < 0.01), in contrast to mannose and M2. Although pH influenced interactions, no significant interaction was detected between pH and enzyme loading. Additionally, the significance of quadratic interaction in each parameter related to M3 generation was emphasized (*p*-value < 0.01).

In the case of M4, pH and temperature exhibited the highest significance (*p*-value < 0.01), followed by enzymatic load. While time was not individually significant, it displayed relevance when interacting with other variables, particularly pH. Moreover, pH and temperature held a high significance value in the model (*p*-value < 0.01), whereas the quadratic enzymatic load was the sole significant factor, consistent with the observed trend in mannose production (*p*-value < 0.01). For M5 production, all variables proved to be significant, with temperature being the least influential. However, interactions related to temperature showed a substantial statistical significance. Time did not exhibit quadratic significance, but the other variables did, with enzymatic load being the most significant (*p*-value < 0.01), as observed in the cases of M3 and M4.

Finally, in the production of the MOS (M2–M5), pH and time were found to be significantly important, while temperature had a lower significance (*p*-value < 0.05). Lastly, quadratic interactions of enzymatic load and temperature were highly significant, with time being of lesser significance. These findings underscore the importance of meticulously analyzing process parameters and their interactions in the production of various compounds, as their influence may vary depending on the specific product being produced. Subsequent paragraphs will delve deeper into the effect of each process variable on mannose and MOS production.

### Influence of the independent parameters in MOS production

**Fig. 1:**
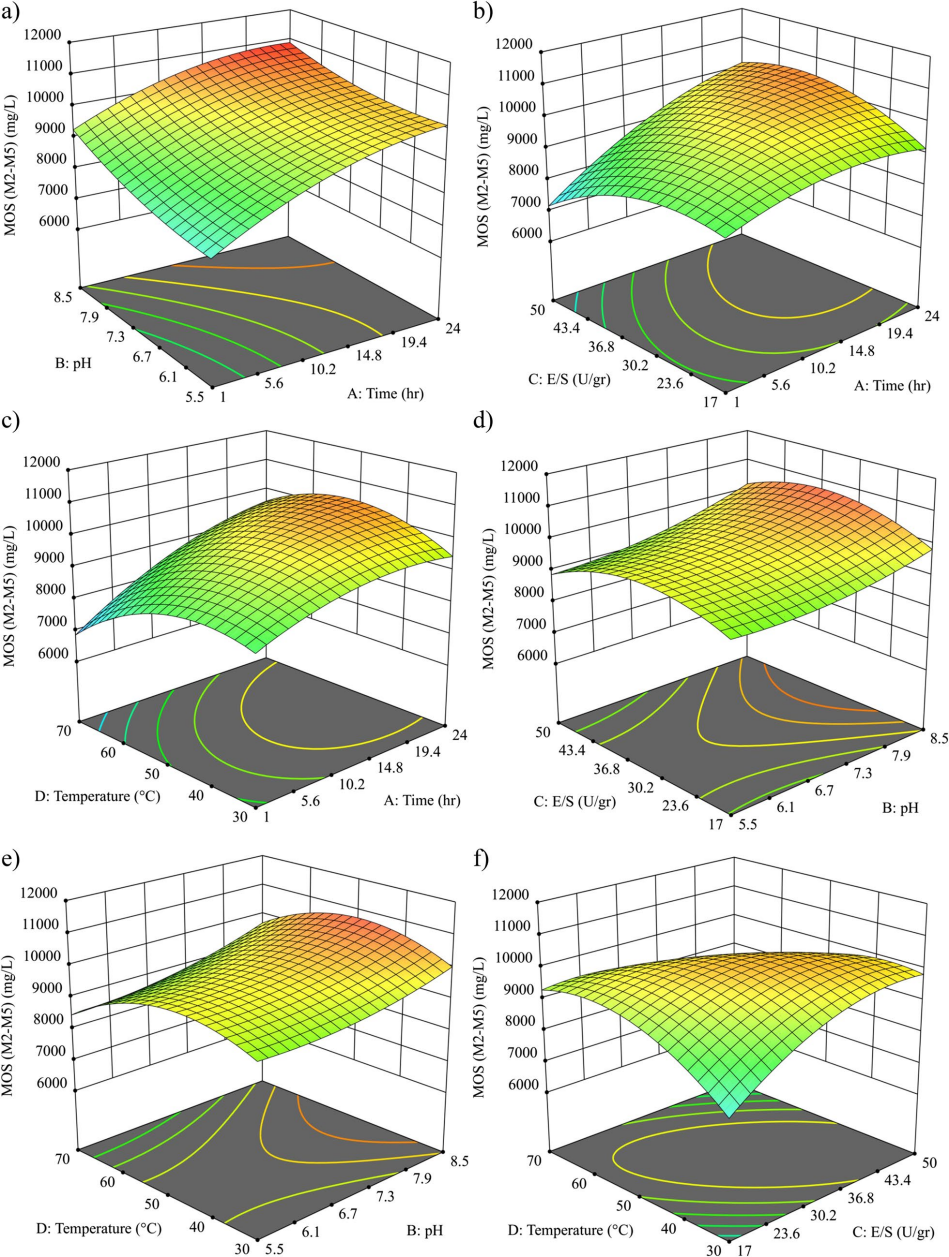
Response surface and corresponding contour curves of the effects of **a** time and pH, **b** time and enzyme loading, **c** time and temperature, **d** pH and enzyme loading, **e** pH and temperature, and **f** enzyme loading and temperature on the concentration of açaí seed–derived MOS (DP 2–5)

In the context of the MOS (M2–M5) response variable, three-dimensional response surface curves and corresponding contour plots are showcased in Fig. 1, presented against two variables. The observed variations in MOS (M2–M5) concentrations ranged from 6 to 10.5 g/L across all considered input variable ranges. This finding suggests a significant conversion of mannan from the sample to MOS (M2–M5) during the early stages of the reaction. This assertion is supported by the observations in Fig. 1, which indicate that the MOS content in the first few hours of reaction accounts for almost half of the total achieved in 24 h, which was almost 11 g/L.

Although, it is important to note that although more than half of the MOS was produced in the first hours, this value corresponds to relatively high concentrations of oligosaccharides with a higher degree of polymerization, such as M4 and M5. Therefore, such a significant variation in concentration is not observed as the largest oligomers are found to be depolymerized to M2 and M3. However, by observing the response surface, it becomes apparent that longer time promotes MOS production, regardless of other influencing factors. This outcome is in line with the intrinsic nature of enzymatic reactions where extending the time increases the interaction between the enzyme and substrate, thereby improving the hydrolysis of mannan in the açaí seed. Moreover, this observation harmonizes with the findings of Intaratrakul et al. (2022), who identified a parallel increase in MOS concentration through enzymatic hydrolysis of defatted copra meal with extended reaction duration, as well as the analogous trends reported by Jian et al. (2013) in the case of *Gleditsia sinensis*.

Furthermore, the pH parameter not only shows significance within the model but also appears to independently influence MOS production. In the pH vs. enzyme loading (Fig. 1d) or pH vs. temperature (Fig. 1e) graphs, such drastic changes are not observed in the behavior of pH, although it is observed that extreme conditions present the highest concentration of MOS. However, in the case of alkaline pH, slightly higher concentrations are reached regardless of the other parameters. This result is consistent with a previous study published by Gonçalves et al. (2023), where their research reports that the use of alkali and the presence of sodium hydroxide increase the MOS production. The alkaline pH implies the presence of hydroxyl ions in the medium, which generates the solubilization of hemicellulose and consequently this polysaccharide is more exposed, facilitating its interaction with hydrolytic enzymes. It should be noted that alkaline pH also favors the solubilization of lignin, which could act as an inhibitor of the enzymes responsible for MOS production (Malgas et al. 2016). However, this effect was not detected in this study, which could be because the percentage of hydrolyzed lignin is below the inhibition threshold of the enzyme.

The utilization of beta-mannosidase enzyme cocktails for the hydrolysis of açaí seed to obtain mannose has been reported at an enzyme loading of 400 U/g of seed (Monteiro et al. 2019). However, for other raw materials aiming to produce MOS from coffee (Arnling Bååth et al. 2018) and *Gleditsia* seeds (Jian et al. 2013), substrate concentrations ranging from 25 to 5 U/g of substrate have been employed. In this context, this study evaluates enzyme concentrations within the range of MOS production and slightly higher. In this regard, a moderate to high concentration evaluated within the enzyme loading ranges (30–50 U/g substrate) proves useful to produce MOS, but factors like pH can assist in reducing this enzyme load, considering the substantial cost associated with enzymes. This behavior suggests the possibility of adjusting enzyme concentrations to a more diluted form while simultaneously maintaining a mildly alkaline environment to boost yields. This approach has the potential to enhance production without incurring substantial enzyme-related expenses. However, it is important to validate this result, as the alkaline environment might favor the production of other compounds, potentially increasing downstream costs due to the need for lignin removal and other subproducts.

The effect of temperature seems to be influenced to a greater extent by the enzyme load than by the other factors, and temperatures oscillating 50 °C can be used to achieve a high concentration of MOS with a less high enzyme load, which also allows positively influencing the costs associated with the enzyme load. Although temperature is closely related to the enzyme used and its origin, this optimal range aligns with the findings reported by Intaratrakul et al. (2022), where enzymatic hydrolysis of copra flour exhibited maximum MOS yield under similar temperature conditions.

### Model verification and optimum reaction conditions

Optimal operating conditions for finding the best hydrolysis conditions for MOS production were obtained using numerical optimization technique. In this study, the goal of optimization was to maximize the production of MOS from M2 to M5 and simultaneously minimize mannose production. Additionally, the objective was to minimize processing time, considering its potential as a variable that could reduce processing duration and, consequently, costs. Table 5 shows the range of values obtained for the optimization process and its purpose.

**Table 5:** Optimization targets and individual and total MOS concentration results range

Variable	Goal	Lower limit	Upper limit
*Time*	Minimize	1	24
*pH*	In range	5.5	8.5
*E/S*	In range	17	50
*Temperature*	In range	30	70
*M1*	Minimize	139.26	1378.11
*M2*	In range	398.68	4424.50
*M3*	In range	1571.77	6416.37
*M4*	In range	548.13	2143.69
*M5*	In range	408.75	3519.53
*MOS (M2-M5)*	Maximize	6342.36	10,984.90

*Lower and upper weight have a value of 1. The importance for all names was 3

Based on the initial solution generated within the software, which yielded a maximum desirability score of 0.867, corresponding to 1 h, a pH of 8.5, an enzyme loading of 35.46 U/g, and a temperature of 37.212 °C. These values closely resemble those observed in Fig. 1, except for temperature. This discrepancy is attributed to the notable increase in mannose production observed at temperatures approaching 50 °C (data not shown).

**Fig. 2:**
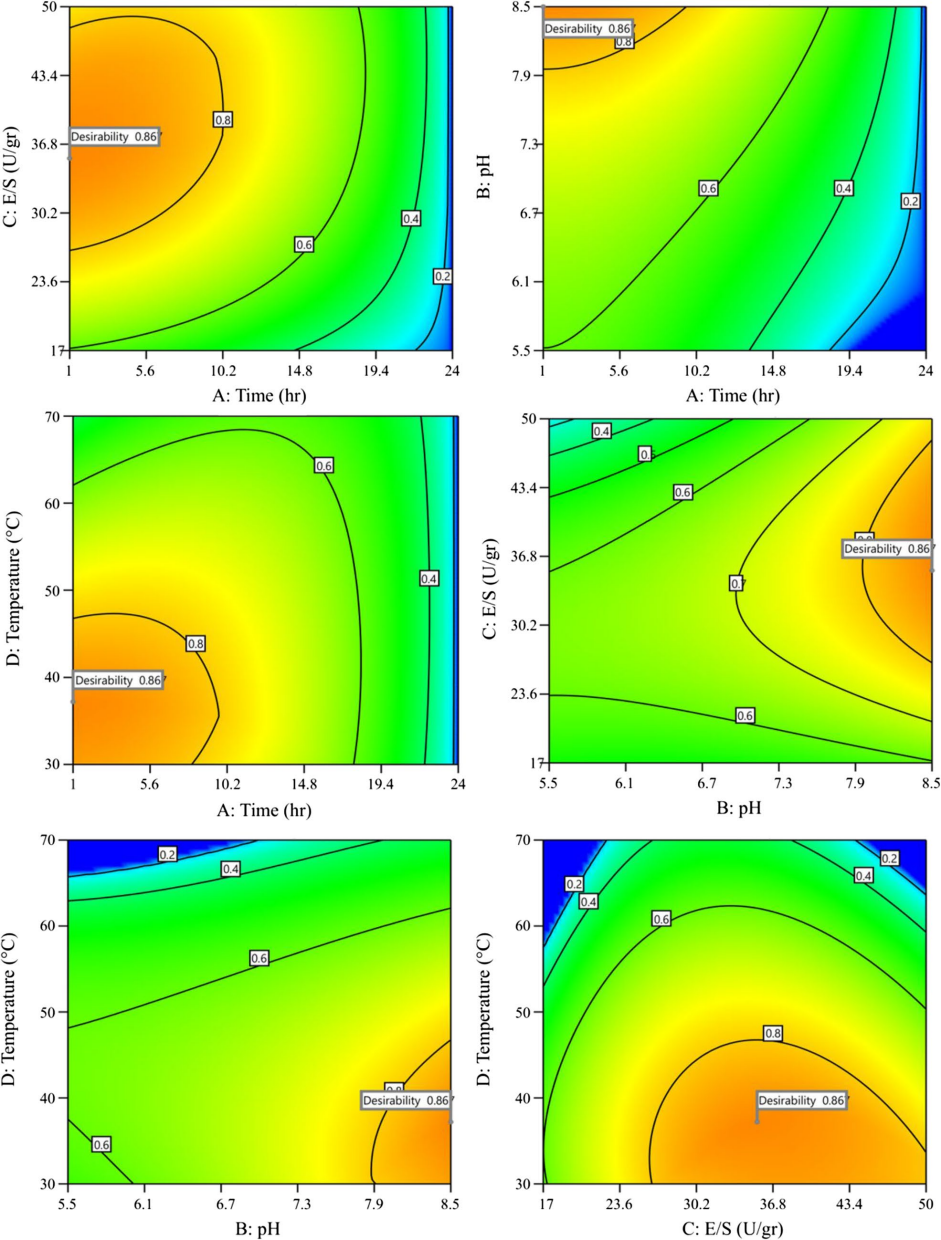
Contour plots of desirability for MOS production

The contour plot of desirability, illustrating the influence of two process variables, is depicted in Fig. 2. It is apparent that MOS production can be achieved within a processing time of 10 h or less by maintaining alkaline conditions and using high or intermediate enzyme loadings. This timeframe aligns with the typical duration for optimizing oligosaccharide production from polysaccharides (de Moura et al. 2015).

The two subsequent best conditions from the numerical optimization, along with the optimized condition mentioned in the preceding paragraphs, were employed to validate the performance of each of the generated models. These conditions are presented in Table 6. The percentage error between the predicted and observed values for various responses was below 9%, indicating the adequacy of the model. Despite conditions 2 and 3 also maximizing the production of MOS and minimizing the production of mannose, they employ longer hydrolysis times, failing to minimize the reaction time unlike condition 1.

**Table 6:** Validation of model with optimized conditions

		Product mg/L	Predicted	Experimented	Error
Condition 1					
		**M1**	333.45	350.45	4.79%
Time (h)	1	**M2**	1760.90	1805.65	2.48%
pH	8.5	**M3**	4130.13	3927.45	5.16%
E/S	35.5	**M4**	1844.72	1901.35	2.98%
Temperature	37	**M5**	2630.38	2615.25	0.58%
		**MOS (M2–M5)**	10,366.14	10,249.71	1.14%
Condition 2					
		**M1**	273.17	287.25	4.90%
Time (h)	2.168	**M2**	1736.56	1698.45	2.24%
pH	8.5	**M3**	4355.70	4487.25	2.93%
E/S	36	**M4**	1832.41	1854.25	1.18%
Temperature	37.5	**M5**	2616.99	2598.45	0.71%
		**MOS (M2–M5)**	10,541.66	10,638.41	0.91%
Condition 3					
		**M1**	175.67	193.25	9.10%
Time (h)	3.094	**M2**	1741.31	1750.63	0.53%
pH	8.5	**M3**	4474.94	4667.39	4.12%
E/S	35	**M4**	1815.71	1810.45	0.29%
Temperature	38.2	**M5**	2633.44	2625.85	0.29%
		**MOS (M2–M5)**	10,665.39	10,854.32	1.74%

The optimized results were compared to established literature data on MOS production through enzymatic hydrolysis in lignocellulosic materials. This findings align closely with previous research: for example, the yield of 14.41 g (M2–M6)/L for copra meal reported by Rungruangsaphakun and Keawsompong (2018), while steam-exploded palm kernel cake yielded 17.4 g (M2–M4)/L, as reported by Li et al. (2018). On the other hand, *Gleditsia sinensis* gum achieved an even higher concentration of 29.1 g (M1–M5)/L, as indicated by Jian et al. (2013). In contrast, copra meal showed lower values at 1.96 g (M2–M6)/L, as reported by Intaratrakul et al. (2022). In this sense, when compared to various materials, the results of our study may be considered modest.

Additionally, the optimized results were recalculated, in terms of MOS yield as explained by Murillo-Franco et al. (2023), yielding 11.34%, 11.54%, and 11.67% per gram of dry açaí seed for conditions 1, 2, and 3, respectively. These values fall within the range reported by the same authors for 24 h, pH 6.5, 50 °C, and 17.5 U/g of seed, which was 10.79% per gram of dry açaí seed; however, the content of oligosaccharides with low polymerization grade was higher than that found in this article. On the other hand, Li et al. (2018) obtained MOS yields ranging from 10.5 to 19.7%, and the oligosaccharide yields of M2–M4 per gram of PKC. However, the PKC was pretreated with steam explosion. In this sense, the results were very promising for açaí seed because they do not require pretreatment compared to a comparable material with pretreatment.

## Future perspectives

In the realm of future research, despite the advancements in obtaining MOS from açaí seeds, it is crucial to explore various hydrolysis alternatives that may surpass the advantages examined in this study. The application of pre-treatments and unconventional methods could potentially offer even more promising solutions than those evaluated here. Additionally, it is imperative to recognize that the purification of MOS in the subsequent processes of the production chain, known for their costliness, requires a strategic approach to achieve high-value products. However, it is necessary to conduct appropriate validations using more specific methods for quantifying açaí seed sugars, such as TFA hydrolysis, alditol acetate derivatization, and gas chromatography, to confirm that the oligomers obtained are exclusively MOS.

Beyond technical validation, comprehensive analyses of economic and environmental feasibility will be necessary to confirm that this alternative can be realized in the real world. Investigating the antioxidant and prebiotic properties of açaí MOS will also be essential for comparing them with MOS derived from other agro-food residues studied previously. These perspectives not only pave the way for their future implementation but also position açaí seeds as a potentially profitable alternative in the realm of food additives.

## Conclusions

This study highlights açaí seeds’ potential for producing mannooligosaccharides (MOS) via enzymatic hydrolysis. Employing response surface methodology (RSM) and quadratic models, it elucidates variable interactions and their impact on MOS yield. Optimizing MOS production, while minimizing mannose content and processing time, carries economic and sustainable implications. Furthermore, it enhances understanding of enzymatic hydrolysis by revealing complex variable interactions. Correlations between enzymatic activity, pH, temperature, and processing time emphasize its intricate nature, aiding in refined process control. Experimental results aligning with model projections validate methodology robustness. Defined optimal parameters—moderate enzyme concentration, alkaline pH, and temperature below 40 °C— prepare the ground for diverse applications, advancing MOS production and impacting food and feed industries.

## Abbreviations

M1: Mannose
M2: Mannobiose
M3: Mannotriose
M4: Mannotetraose
M5: Mannopentaose
M6: Mannohexose
MOS: Mannooligosaccharides
